# Deer Rescue in Tuscany: Retrospective Analysis and Assessment of Radiography Diagnoses

**DOI:** 10.3390/ani11113087

**Published:** 2021-10-29

**Authors:** Irene Nocera, Caterina Puccinelli, Micaela Sgorbini, Simone Scoccianti, Marco Aloisi, Claudia Biliotti, Simonetta Citi

**Affiliations:** 1 Department of Veterinary Sciences, University of Pisa, 56122 Pisa, Italy; irene.nocera@vet.unipi.it (I.N.); caterina.puccinelli@phd.unipi.it (C.P.); simonetta.citi@unipi.it (S.C.); 2 H24 Vet Hospital, 50142 Florence, Italy; s.scoccianti@vethospital.it; 3 C.R.A.S.M. (Maremma Wild Animal Rescue Center), 58055 Semproniano, Italy; marcoaloisi@yahoo.it (M.A.); biliotti.cla@gmail.com (C.B.)

**Keywords:** wildlife emergencies, wildlife rescue, ungulates, row deer, fallow deer, radiography, Tuscany

## Abstract

**Simple Summary:**

Animal-vehicle collisions are the major cause of rescue and need for hospitalization in wildlife referral centers. Radiography is used to assess the traumatized animal and is a rapid means to evaluate various traumatic pathologies. Clinicians can exploit radiography when making rapid decisions about whether to euthanize or treat an animal. We evaluated data (reasons for rescue, diagnosed lesions, and outcome) from a population of hospitalized wildlife ungulates and we investigated the benefits of using radiography as a diagnostic tool.

**Abstract:**

Animal-vehicle collisions are the major cause of rescue and need for hospitalization in wildlife referral centers. Clinicians need easy-to-use tools to make rapid decisions about whether to euthanize or treat injured animals. The aim of the study was to evaluate the data (reasons for rescue, diagnosed lesions, and outcome) from a hospitalized population of wildlife ungulates and investigate the benefits of using radiography as a diagnostic tool. Data were collected from three wildlife referral centers in Tuscany (Italy). The following information was collected for each animal: reason for hospitalization, clinical examination, radiographic examination, definitive diagnosis, and outcome. A chi-squared test was used to assess the benefits of radiography in detecting different traumatic lesions. Prevalence was reported according to the reason for hospitalization, definitive diagnosis, radiographic diagnosis, and outcome. The main reason for hospitalization was traumatic lesions due to vehicle collisions and 71.1% of the animals did not survive. Radiography was more useful in patients with traumatic axial skeletal lesions and/or multiple traumas with respect to traumatic appendicular skeleton lesions. Our results show that radiography is a useful diagnostic technique for assessing wildlife emergencies and it could help the clinician in making medical decisions.

## 1. Introduction

In the last few decades, the population of wildlife ungulates has increased exponentially in Western Europe [1,2], including Italy, where our study took place [3,4]. This should be seen as part of a wider and general phenomenon, related to deep changes in the environment where these species live and interact with the human population [1].

In Tuscany (central Italy), this increase was the result of the reduction in agricultural practices in hilly and mountainous areas, along with an increase in woodland or forested areas, changes in agricultural practices (i.e., the proliferation of winter cereals), less live-stock husbandry, changes in hunting and management (including controlled culling and reintroduction) practices, and a warm climate [5]. The increase in the wildlife ungulates population led to the expansion of animals in nearby areas leading to more interactions between humans and wildlife ungulates [5].

Tuscany has wide expanses of rural areas crossed by extensive road networks and urban areas. The conflict between wildlife and human activities has led to a sharp rise in deer–vehicle collisions [3,4,6,7,8,9]. Some studies have shown that cervid movement is the main factor influencing collision risk and frequency, but also that deer–vehicle accidents are related to habitat, climatic, and traffic characteristics, as well as predation, hunting, and disturbance effects [10,11,12,13].

The animal-vehicle collisions represent a direct cause of death for wildlife mammals every year, and the main cause of wildlife rescues and admission to specialized veterinary hospitals (VHs) for first aid [3,4], with a wide range of different species involved [13,14,15,16]. Animal-vehicle collisions not only affect wildlife populations, but also endanger humans [17]. Deer-vehicle collisions have been associated with ecological, social, and economic consequences, such as property damage, deer loss, and human injury and death [17].

The specialized wildlife referral centers rescue and provide first aid to injured animals. To ensure the welfare of individuals, a proven process needs to be followed that enables the clinician to make a rapid decision about euthanasia or clinical recovery of animals [6,7,8,9]. The decisions should be made quickly, ideally within 48 h of admission, in order to prevent unnecessary suffering or casualties in captivity [6,7,8,9].

An accurate clinical general veterinarian examination represents the starting point and sometimes it is followed by laboratory examinations, radiology, and ultrasonography [6,7,8,9]. Only approximately 40% of wildlife casualties, across all species and ages, are suitable for release back into the wild. Although radiology is frequently used to assess traumas in small animals [18], and several studies have evaluated its utility for various traumatic pathologies [19,20,21,22,23], to the best of our knowledge, there have been no studies on using radiology in wildlife ungulates to assess traumatic injuries.

The aims were: (1) to assess data collected by three wildlife referral centers on the reason for rescue, the diagnosed lesions, and the outcome of a cohort of roe deer and fallow deer in Tuscany (Italy); (2) to assess the benefit of using radiology as a diagnostic tool during emergencies, investigating the feasibility of using the clinical diagnosis alone or associated with radiology.

## 2. Materials and Methods

### 2.1. Data Collection

In this retrospective study, medical records for 2015–2020 were collected and analyzed from a cohort of rescued roe deer (*Capreolus capreolus*) and fallow deer (*Dama dama*), from three centers in Tuscany (Italy) specializing in giving first aid to wildlife. The deer were rescued in multiple areas of Tuscany (Pisa, Grosseto, Siena, Florence, and Arezzo), and from different municipalities. Details on rescue areas are reported in Table 1.

The following information was included in the survey: (1) reason for hospitalization, (2) outcome, (3) clinical diagnosis, and (4) radiographic diagnosis, if performed. When radiographic examination was needed, it was performed under sedation or general anesthesia in a clinical setting in order to reduce the stress and handling time. All the animals that had undergone a radiographic examination were assessed in two of the rescue centers involved in the study. The radiographs were acquired using a high-frequency digital radiography system (MAXIVET 400 HF, Multimage s.r.l., Cavaria, Varese, Italy and Ida 9 G, ISOMEDIC, Somaglia, Italy). The clinical examinations and radiographs were performed under sedation or general anesthesia to reduce the stress and handling time. The same anesthetic protocol was followed in both the rescue centers. Deep sedation was obtained by the association of dexmedetomidine 8 ± 1.3 mcg/kg, ketamine 2 mg/kg, and midazolam 0.2 mg/kg given intramuscularly. During sedation, mask oxygen was administered to all the subjects. General anesthesia was performed using propofol 2 mg/kg IV, and endotracheal intubation was performed only in the case of respiratory depression.

Based on the reason for hospitalization, the lesions of the deer were grouped as follows: (1) vehicle collisions (certain or assumed), (2) entrapment in nets/fences, (3) combine harvesters, gunshot, and predation.

Based on the outcome, the deer were classified as follows: (1) survived (released/given custody), (2) died (spontaneously/euthanized), (3) unknown.

According to the clinical diagnosis and the radiographic diagnosis (if performed), the deer were grouped into the categories shown in Table 2.

### 2.2. Statistical Analysis

The prevalent reason of hospitalization, the outcome, the clinical diagnosis, and radiographic diagnosis (if performed) were identified for each category of roe deer, fallow deer, and the total number of animals enrolled in the study.

In order to verify the feasibility of using the clinical diagnosis alone or associated with radiology, the animals that had undergone both clinical and radiographic diagnosis were divided into two sub-groups: (a) sub-group A: traumatic skeletal injuries of the appendicular skeleton: in this group, forelimb and/or hindlimb fracture/luxation were included; (b) sub-group B: traumatic skeletal injuries of the axial skeleton and/or multiple trauma: in this group, vertebral fracture/luxation, pelvic fracture/diastasis, and multiple traumas were included. Furthermore, a chi-squared test was used to verify the differences between the two groups. The statistical analysis was performed using Graph Pad Prism (San Diego, CA, USA), and the significance was set at *p* < 0.05.

## 3. Results

A total of 1135 records were assessed, of which 1070/1135 (94.3%) were roe deer and 65/1135 (5.7%) were fallow deer. 

The main reason for hospitalization was traumatic lesions due to vehicle collision (certain or assumed) both in roe and fallow deer, as shown in Table 3.

Regarding the outcome, most of the roe and fallow deer died spontaneously or were euthanized, as shown in Table 4.

Clinical diagnosis pointed out a higher prevalence of multiple trauma both in roe and fallow deer, followed by the other traumatic skeletal lesions, as described in Table 5.

X-rays were performed in 163 out of 1135 (14.4%) ungulates, of which 145/163 (89.0%) were roe deer and 18/163 (11.0%) were fallow deer. In 121/163 animals, the radiographic exam highlighted traumatic skeletal lesions, whereas in 42/163 patients, no traumatic skeletal lesions were detected. Table 6 shows the results of the prevalence of the radiographic diagnosis in the ungulates with traumatic skeletal lesions. The results show a similar distribution within categories, but forelimb fracture/luxation in roe deer was the least represented 8/145 (5.5%) and hindlimb fracture/luxation in fallow deer had the highest prevalence 5/18 (27.8%).

Table 7 shows the results of the prevalence of the outcome in the ungulates with traumatic skeletal lesions. Most of the roe and fallow deer died spontaneously or were euthanized; within survived ungulates (8/121, 6.6%), 7/8 were multiple traumatic ungulates (2/7 fallow deer and 5/7 roe deer), and 1/8 hindlimb luxation in roe deer.

Table 8 shows the results on the accordance between clinical and radiographic diagnosis in the group A and B, expressed as number and proportions (n/%) of the roe deer, fallow deer, and total population.

**Figure 1:**
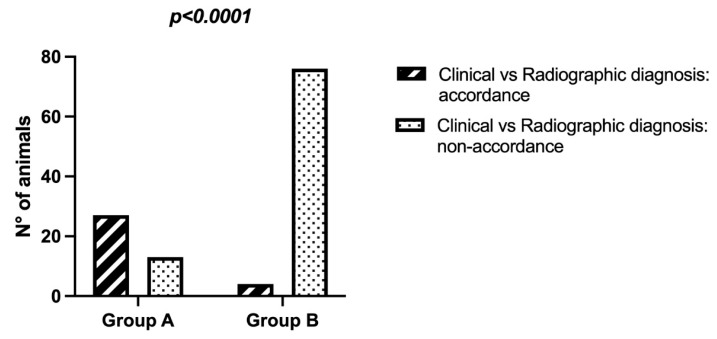
Chi-squared test between groups A and B, considering the accordance or non-accordance between clinical and radiographic diagnosis.

A chi-squared test showed statistically significant differences (*p* < 0.0001) between groups A and B (Figure 1), considering the accordance or non-accordance between clinical and radiographic diagnosis.

## 4. Discussion

Our retrospective study analyzed the data collected from a cohort of 1135 roe deer and fallow deer admitted to three rescue centers. The first aim was to assess the reason for rescue, the clinical and radiographic diagnosis (if performed), and the outcome.

Our results showed that the main reasons for rescue and hospitalization were traumatic injuries and the most represented was trauma caused by certain or assumed collisions with vehicles. A study about rescued roe deer carried out in Emilia-Romagna (Italy) showed similar findings, with a prevalence of 71.4% of patients hospitalized for trauma [24]. Our results are in line also with previous reports [3,4] in which the prevalence for rescue was evaluated in a more limited geographical area in Tuscany (municipality of Pisa). Pacini and colleagues [4] reported a prevalence of 71% of deer emergencies due to road accidents.

Our results are also in line with studies in other countries in Europe [25,26,27]. Accidents involving roe deer represent the majority of the wildlife collisions with vehicles in Lithuania [25,26] and Poland, where over half of the traffic incidents (66%) involving wildlife were collisions with roe deer [27]. Additionally, in a study performed in Switzerland about causes of mortality and morbidity in roe deer, the main diagnoses of non-infectious problems were traumas (61%), including blunt trauma due to traffic accidents [28]. In the UK, animal-vehicle collisions represented 37% of the adult badger casualties admitted to wildlife hospitals [29].

Several authors have underlined that animal–vehicle collisions are usually unreported, and that accurate records are lacking [24,30,31,32], leading to an incorrect evaluation and monitoring of the current situation. Moreover, different ecological factors, such as density, areas with different landscapes, climates, and population structures influence the probability of deer having a car accident [33].

Recording the number of car accidents involving animals combined with the numbers of rescued wildlife would allow the monitoring of the wildlife population [33]. Thus, in this light, our study could be used to promote surveillance and monitoring as part of national and international wildlife health surveys.

Regarding the clinical diagnosis, the majority of deer enrolled in this study were affected by traumatic skeletal injuries and/or multiple traumas. This finding is in line with previous studies [34,35] which reported skeletal fractures as being the most common traumatic injuries, in particular related to animal–vehicle collisions [35].

Most of the animals hospitalized died spontaneously or were euthanized (71.9% of roe deer and 69.2% of fallow deer). Our results are in line with previous studies that reported high mortality of rescued wildlife animals [4,30] and a low number of subjects suitable for release back into the wild (40%) [36].

Our results on the outcome are likely related to the reason for hospitalization, in line with others [2,34]. In fact, the deer population evaluated in this study was mostly affected by severe traumatic injuries caused by vehicle collisions. In our study, radiographic diagnosis identified severe traumatic skeletal lesions (e.g., vertebral fracture/luxation, hindlimb/front limb fracture) that could not be successfully treated and therefore with no possibility of full rehabilitation, and thus the best option was euthanasia [37]. Only a small group of ungulates, mostly affected by multiple trauma (6.6%), were suitable for release back into the wild.

In one of the three centers included in the study, the medical records were sometimes incomplete; thus, it was not possible to know the outcome of all the patients included, and, in these cases, the outcome was classified as “unknown”. This could represent a limit for the study.

Our second aim was to verify the effectiveness of radiography compared to clinical diagnosis alone. We did not evaluate the impact of performing a radiographic examination on the outcome. Clinicians need to make rapid decisions about whether to euthanize or hospitalize animals [38,39]. The decisions should be made quickly, ideally within 48 h from admission, in order to prevent subsequent unnecessary suffering in captivity [38,39]. An accurate veterinarian examination is the starting point and is sometimes followed by laboratory tests, radiology, and ultrasonography examination [40].

In small animals, radiography is frequently used to assess veterinary traumatized patients [18]; however, its utility has not been studied in wildlife ungulates. Our findings showed that radiography is more useful in animals affected by traumatic axial skeletal lesion and/or multiple trauma (group B) with respect to the traumatic appendicular skeleton lesions and traumas (group A). We found that the non-agreement between clinical and radiographic diagnosis was 95.1% in group B and 32.5% in group A. This finding agrees with a study performed in feline trauma patients in which whole-body radiographs were used to detect thoracic, abdominal, pelvic, and spinal injury [22]. Appendicular skeleton lesions could, perhaps, be diagnosed with a clinical evaluation alone (severe lameness, swelling, pain, deformity, abnormal mobility, or crepitus at the affected site) because the affected site is easier to localize and assess [41].

Thoracic and abdominal ultrasound (US), and computed tomography (CT) are also useful diagnostic imaging techniques for traumatized animals [42,43,44,45]. The US assessment of the thorax and abdomen is reported to be a rapid and accurate method to detect traumas in dogs [42]. CT is considered the gold standard for the evaluation of acute canine spinal trauma [44]. It also appears to be more sensitive than ultrasound and radiography in the identification of thoracic pathologies in traumatized patients (i.e., pleural fluid, pulmonary contusion), but further studies are needed [45]. Thoracic or abdomen US or CT were not performed in the ungulates enrolled in this study; thus, a comparison between different diagnostic imaging procedures for the diagnosis of traumas was not possible.

In other European countries, trauma has been described as one of the major causes or contributing causes of death in roe deer [46,47,48], and clinicians need to make a rapid decision about the euthanasia or clinical recovery of these animals [38,39]. Based on our results, radiography can help to identify traumatic lesions of the spine, of the pelvis, or multiple skeletal traumas, which indicate that the animal will probably not have a reasonable chance of survival upon release. Regarding the traumatic lesions of the appendicular skeleton, our results showed that a clinical evaluation could be sufficient for the diagnosis; however, in our opinion, radiography helps to correctly classify the type of lesion (e.g., fracture vs. luxation) and is essential for orthopedic surgery [49].

## 5. Conclusions

Our results indicate that radiography examination is a useful diagnostic technique for assessing pathologies that are not clinically evident in rescued wild animals. We believe that the use of radiography is essential in deer emergencies with a history of traumatic injuries and is a key means to make a diagnosis and rapidly decide on the best treatment. 

## Figures and Tables

**Table 1:** Rescue areas details covered by the survey.

Province	Roe Deer (n/%)	Fallow Deer (n/%)	Total (n/%)
Pisa	53/1070 (5%)	13/65 (20%)	66/1135 (5.8%)
Grosseto	152/1070 (14.2%)	19/65 (29.2%)	171/1135 (15.1%)
Siena	510/1070 (47.6%)	15/65 (23.1%)	525/1135 (46.3%)
Florence	320/1070 (29.9%)	12/65 (18.5%)	332/1135 (29.2%)
Arezzo	35/1070 (3.3%)	6/65 (9.2%)	41/1135 (3.6%)

**Table 2:** Categories for clinical diagnosis classification.

Diagnosis Classification		
Traumatic skeletal lesions	Forelimb fracture/luxation	one or more fractures and/or articular luxation affecting the bones of one or both forelimbs
	Hindlimb fracture/luxation	one or more fractures and/or articular luxation affecting the bones of one or both hindlimbs
	Vertebral fracture/luxation	one or more fractures and/or luxation affecting one or more vertebrae
	Pelvic fracture/diastasis	one or more fractures of the bones of the pelvis and/or diastasis of the pelvic symphysis
	Multiple trauma	simultaneous presence of clinically significant injuries to multiple body regions or cavity, compromising the animal’s physiology, including pneumothorax, lung contusion and/or rib fractures
Other traumatic lesions	Traumatic shock	animals with clinical signs of hypovolemic shock and/or signs of organ dysfunction due to a traumatic event
	Wounds	superficial, deep, or penetrating wounds
	Paraplegia	hindlimb paralysis
	Tetraplegia	forelimb and hindlimb paralysis
	Head lesions	head trauma: neurological clinical signs of traumatic brain injury skull trauma: one or more fractures of the cranial bones horn base fracture

**Table 3:** Reasons for hospitalization expressed as the number and proportions (n/%) of roe deer and fallow deer, and total population.

Reason for Hospitalization	Roe Deer (n/%)	Fallow Deer (n/%)	Total (n/%)
Lesions due to vehicle collision (certain or assumed)	990/1070 (92.6%)	56/65 (86.1%)	1046/1135 (92.2%)
Lesions due to being trapped in nets/fences	52/1070 (4.8%)	7/65 (10.8%)	59/1135/5.2%)
Lesions due to combine harvesters/gunshot/predation	28/1070 (2.6%)	2/65 (3.1%)	30/1135 (2.6%)

**Table 4:** Outcome expressed as the number and proportions (n/%) of roe deer and fallow deer, and total population.

Outcome	Roe Deer (n/%)	Fallow Deer (n/%)	Total (n/%)
Survived	203/1070 (19.0%)	14/65 (21.5%)	217/1135 (19.1%)
Dead	770/1070 (71.9%)	45/65 (69.2%)	815/1135 (71.8%)
Unknown outcome	97/1070 (9.1%)	6/65 (9.3%)	103/1135 (9.1%)

**Table 5:** Clinical diagnosis expressed as the number and proportions (n/%) of roe deer, fallow deer, and total population.

Clinical Diagnosis		Roe Deer (n/%)	Fallow Deer (n/%)	Total (n/%)
Traumatic skeletal lesions	Forelimb fracture/luxation	78/1070 (7.8%)	6/65 (9.2%)	714/1135 (62.9%)
	Hindlimb fracture/luxation	153/1070 (14.3%)	8/65 (12.3%)	
	Vertebral fracture/luxation	103/1070 (9.6%)	3/65 (4.6%)	
	Pelvic fracture/diastasis	46/1070 (4.3%)	7/65 (10.8%)	
	Multiple trauma	290/1070 (27.1%)	20/65 (30.7%)	
Other traumatic lesions	Traumatic shock	171/1070 (16.0%)	4/65 (6.1%)	421/1135 (37.1%)
	Wounds	25/1070 (2.3%)	3/65 (4.6%)	
	Paraplegia	24/1070 (2.3%)	3/65 (4.6%)	
	Tetraplegia	1/1070 (0.1%)	-	
	Head lesions	179/1070 (16.7%)	11/65 (16.9%)	

**Table 6:** Radiographic diagnosis expressed as the number and proportions (n/%) of roe deer, fallow deer, and total population.

Radiographic Diagnosis		Roe Deer (n/%)	Fallow Deer (n/%)	Total (n/%)
Traumatic skeletal lesions	Forelimb fracture/luxation	8/145 (5.5%)	2/18 (11.1%)	121/163 (79.1%)
	Hindlimb fracture/luxation	25/145 (17.2%)	5/18 (27.8%)	
	Vertebral fracture/luxation	26/145 (17.9%)	3/18 (16.7%)	
	Pelvic fracture/diastasis	21/145 (14.5%)	3/18 (16.7%)	
	Multiple trauma	26/145 (17.9%)	2/18 (11.1%)	

**Table 7:** Outcome in the ungulates with traumatic skeletal lesions, expressed as the number and proportions (n/%) of roe deer and fallow deer, and total population.

Outcome	Roe Deer (n/%)	Fallow Deer (n/%)	Total (n/%)
Survived	6/106 (5.6%)	2/15 (13.3%)	8/121 (6.6%)
Dead	98/106 (92.5%)	13/15 (86.7%)	111/121 (91.7%)
Unknown outcome	2/106 (1.9%)	0/15 (0%)	2/121 (1.7%)

**Table 8:** Accordance or non-accordance between clinical and radiographic diagnosis in groups A and B, expressed as the number and proportions (n/%) of the roe deer, fallow deer, and total population.

		Clinical vs. Radiographic Diagnosis	
Categories	Wildlife Ungulates	Accordance	Non-Accordance
Group A	Roe deer (n/%)	21/33 (63.6%)	12/33 (36.4%)
	Fallow deer (n/%)	6/7 (85.7%)	1/7 (14.3%)
	Total (n/%)	27/40 (67.5%)	13/40 (32.5%)
Group B	Roe deer (n/%)	3/73 (4.1%)	70/73 (95.9%)
	Fallow deer (n/%)	1/8 (12.5%)	7/8 (87.5%)
	Total (n/%)	4/81 (4.9%)	77/81 (95.1%)

Legend—Group A: traumatic skeletal injuries of the appendicular skeleton; Group B: traumatic skeletal injuries of the axial skeleton and/or multiple traumas.

## Data Availability

The data presented in this study are available on request from the corresponding author.
